# Multielement analysis coupled with chemometrics modelling for geographical origin classification of teff [*Eragrostis tef (Zuccagni) Trotter*] grains from Amhara Region, Ethiopia

**DOI:** 10.1186/s13065-023-00972-9

**Published:** 2023-06-08

**Authors:** Chaltu Reta, Tihitinna Asmellash, Minaleshewa Atlabachew, Bewketu Mehari

**Affiliations:** 1 Department of Chemistry, College of Science, Bahir Dar University, P.O.Box 79, Bahir Dar, Ethiopia; 2 Department of Chemistry, College of Natural and Computational Sciences, University of Gondar, P.O.Box 196, Gondar, Ethiopia

**Keywords:** Teff, Multielement, Geographical origin, Multivariate, Amhara, Ethiopia

## Abstract

Teff [*Eragrostis tef (Zuccagni) Trotter*] is an indigenous crop in Ethiopia, and Amhara region is the predominant teff producing region in the country. This study was aimed at developing an analytical methodology useful to determine the geographical origin of teff produced in the Amhara Region, based on multielement analysis combined with multivariate statistical techniques. For this, a total of 72 teff grain samples were collected from three zones (West Gojjam, East Gojjam, and Awi) and analysed for K, Na, Mg, Ca, Mn, Cu, Fe, Co, Ni, Zn, Cr, and Cd contents using inductively coupled plasma-optical emission spectroscopy (ICP-OES). The digestion and ICP-OES analysis method were accurate, with percentage recovery ranging 85.5 to 109% across the different metals analysed. Principal component analysis (PCA) and linear discriminant analysis (LDA) were applied to discriminate samples based on their production regions. Magnesium, Ca, Fe, Mn, and Zn were the most discriminating elements among the samples. The LDA model provided 96% correct classification of samples into production regions and varietal types, with an average prediction ability of 92%. Hence, the multielement analysis combined with statistical modeling can be used in the authentication of the geographical origin and varietal type of teff from Amhara region.

## Introduction

Teff [*Eragrostis tef (Zuccagni*) *Trotter*] is a native cereal grain to Africa and is used as a staple crop in Ethiopia, Eritrea, Djibouti, Sudan's south-eastern region, and northern Kenya [1]. Teff is the most intensively produced cereal grain for human consumption in Ethiopia, followed by maize [2]. In comparison to other cereals like wheat, rice, oat, and barley, teff has a higher nutritional and fibre contents [3, 4]. Teff is a gluten-free cereal grain, and hence it is an ideal substitute for wheat and other cereals in food products [5, 6].

Teff is a warm seasonal grain that can be grown in wide climate conditions, such as in temperatures ranging from 10 to 27 °C and altitudes up to 2800 m above sea level [3, 7, 8]. In Ethiopia, teff covers about 2.8 million hectares, accounting for 25–30% of the total cereal acreage in the country [9, 10].

Amhara region is one of the major teff producer regions in the country. Within the region, East Gojjam, West Gojjam, and Awi zones are known for cultivating a significant amount of teff, and contributing the major share of the region’s teff production quantity. Although several varieties of teff are registered and available in the country, only three varieties are cultivated widely in the three zones. These are white teff, which includes *Kuncho* (DZ-CR-387) and *Tseday* (DZ-CR-37) varieties, red teff, locally called “*Qey teff*”, and mixed or ivory-colored teff, locally called “*Sergegna teff*”. The white Kuncho variety is mostly farmed in East and West Gojjam zones, while the white Tsedey, Qey, and Sergegna teff varieties are widely grown in Awi zone. The Kuncho and Tseday teff types are white in color and indistinguishable by visual observation.

In Ethiopia, teff grains are marketed by the names of their cultivation zones and districts. Consequently, the price depends on the cultivation region and varietal type [11, 12]. For example, white teff from Bichena district of East Gojjam zone is the most appreciated teff by consumers and is more expensive than others. However, inferior teff types are mislabelled and sold with the name of Bichena teff. Thus, a simple method that distinguishes the cultivation region of teff is in high demand to protect against fraudulent activities and mislabeling of inferior teffs in the market. It has to be noted that mislabelling would negatively affect both the consumer, farmer and legitimate merchant. In this respect, it is important to establish methods that facilitate recognition of the geographical origin of teff to protect both consumers and producers.

Multi-element analysis has been effectively used to discriminate the geographical origin of various food products and provide information about the cultivation environment [13, 14]. The discrimination of the geographical origin of various foodstuffs has been achieved using a combination of multivariate data analysis tools and the diverse elemental contents of food products. For instance, cereal grains such as rice, wheat, soybean, millet, tea, and coffee were classified to their respective region of origin based on their elemental composition together with some chemometrics models [15–20].

Thus, this study aimed to determine the levels of metals in teff grain from 21 sub- districts of three zones (East Gojjam, West Gojjam, and Awi zones) of Amhara region of Ethiopia by using inductively coupled plasma optical emission spectrometry (ICP-OES). The study also attempted to determine the variabilities of metal contents across the zones and evaluate the adequacy of the muti-element data in predicting the terroir of teff grains.

## Chemicals, reagents and materials

### Chemicals and reagents

The reagents HClO_4_ (70%) and HNO_3_ (69–72%) (Blulux,India) were used for the digestion of teff grains. CPI International, ICP quality control standards of multielements (USA) of K, Ca, Na, Fe, Mg, Mn, Zn, Ni, Cu, Cr, Co, and Cd were used in the study. The standard working solutions of the metals were prepared freshly from the stock standard solution.

### Materials

Kjeldahl system (Velp Scientifica, Italy) was used for the digestion of teff samples. Inductively coupled plasma optical emission spectrometry (Perkin Elmer, Optima 8000) was used for the determination of multi-elements.

### Sample collection and pre-treatment

**Fig. 1:**
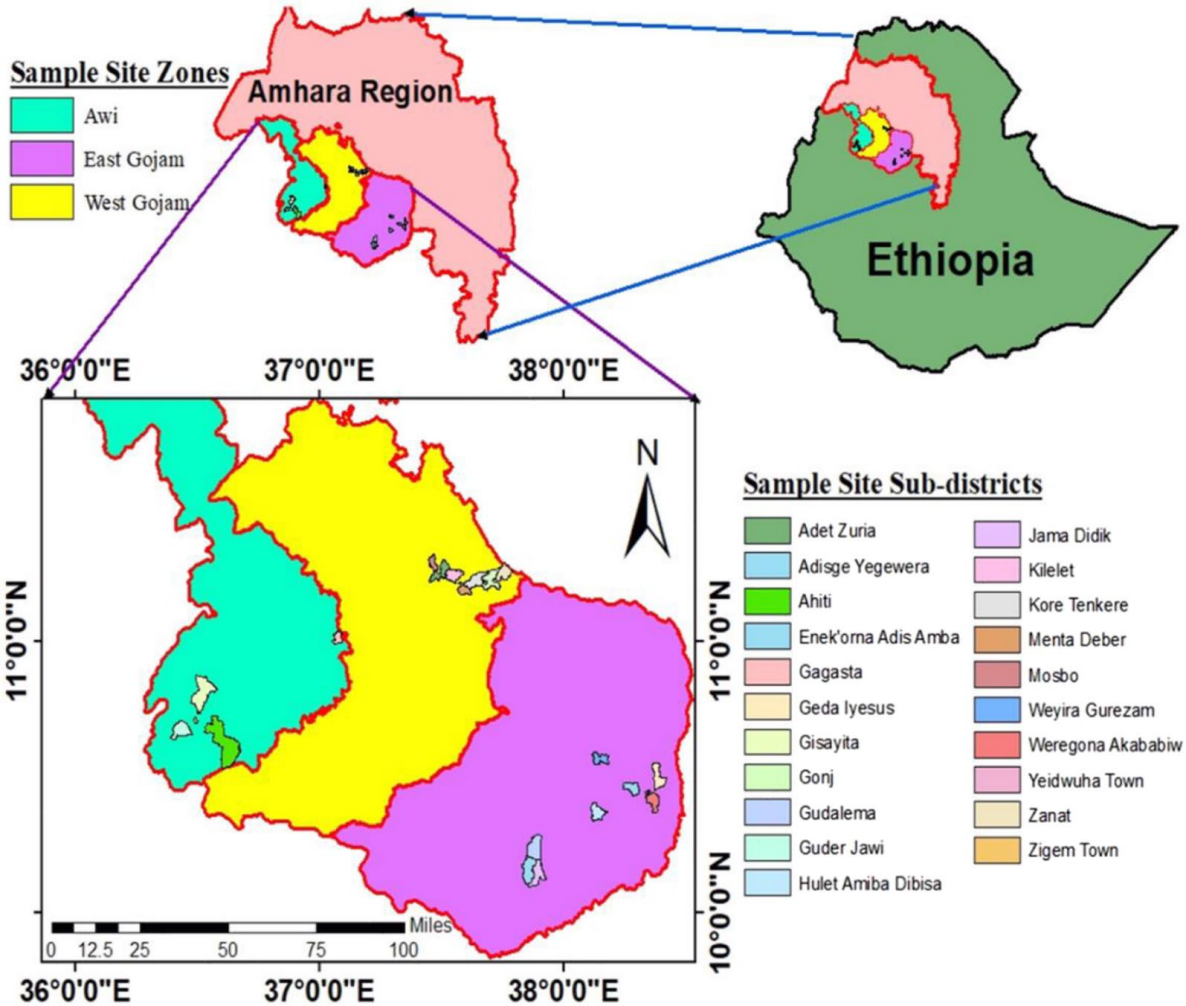
Map of Amhara Region showing teff sampling zones and sub-districts. Source Reta et al. [21], published in Plos One, where the journal applies the Creative Commons Attribution (CC BY) license to make articles legally available for reuse, without permission or fees, for virtually any purpose

During the 2020 harvest season, seventy two teff samples were directly collected from farmers in the three administrative zones (East Gojjam, West Gojjam, and Awi zones) of Amhara region, Ethiopia. Based on the information obtained from Amhara Region Bureau of Agriculture, these three zones are the country's most significant teff producing zones, which contribute voluminous teff grains to the market. From each zone, major teff producing districts and sub-districts were selected for sampling (Fig. 1). From East Gojjam zone, three districts (Aneded, Shebelberenta, and Enemay) and nine sub-districts (Jama Didik, Gudalema, Adisge Yegewera, Yeidwuha Town, Weregona Akababiw, Geda Iyesus, Weyira Gurazam, Hulet Amiba Dibisa, and Enekorna Adis Amba) were selected. From West Gojjam zone, two districts (Goinj kolela and Adet/Yilmanadensa) and seven sub-districts (Zanat, Gonj, Kore Tenkere, Menta Deber, Kilelet, Adet Zuria, and Mosbo) were selected. Where, five sub-districts (Gisayita, Gagasta, Ahiti, Zigem Town, and Guder Jawi) were chosen from Zigem district of Awi zone.

From each sampling site, 500 g of teff grains were collected.The samples were washed with tap water,rinsed twice with distilled water, and further rinsed with deionized water to eliminate adsorbed dust and particulate matter. The samples were oven dried for 12 h to remove moisture and obtain constant mass. The dried samples were ground using an electric grinder, sieved with a 0.5 mm pore-sized sieve and stored in bottles under airtight conditions until digestion and analysis.

### Optimization of digestion procedure

Wet acid digestion is one of the methods involved in getting free metal ions in dissolved form from complex organic matrix. The digestion procedure was optimized by changing different parameters like volume ratio of reagents, temperature, and time (Table 1). The acid digestion was carried out by using Kjeldahl digestion system. It was assumed that digestion is complete when the solution becomes clear and colourless.

**Table 1:** Reagent volume, temperature and time attempted during optimization of digestion of 0.5 g of teff sample

Volume of reagent (mL)		T (^o^C)	Time (h)	Observation
HNO_3_	HCLO_4_			
5	1	160	2:00	Dried residue
3	3	160	2:00	Clear and colourless solution*
4	2	160	2:00	Yellowish precipitate
2	4	160	2:00	Cloud yellow
1	5	160	2:00	Slightly colourless

^*^The optimimum digestion condition

### Instrument calibration and sample analysis

Ten working standard solutions that contain the elements (Na, K, Mg, Ca, Cr, Mn, Fe, Co, Ni, Cu, Zn, and Cd) were prepared and used to calibrate the ICP-OES before the analysis of samples.The concentrations of working standard solutions were 0.05, 1, 2, 4, 6, 8, 10, 12, 14, and 16 mg/L for Cr, Mn, Co, Ni, Cu, Zn, and Cd while for Na, K, Mg, Ca, and Fe were 0.05, 1, 10, 20, 30, 40, 50, 60, 70, and 80 mg/L. A single measurement was performed for the calibration standards and the recovery experiment, while triple measurements were carried out on the digested teff samples. The accuracy of the method was assessed through recovery experiments, where samples were spiked with a known concentration of the standard solution of each metal and digested and analysed following the optimized procedure.

### Method detection and quantification limits

For the determination of the limit of detection (LOD) of the analytical method, the standard deviation (SD) of the blank signals were multiplied by three and devided by the slop of the calibration equations. The limit of quantification (LOQ) was calculated as ten times the standard deviation of the blank devided by the slop of the calibration equations [22]. The wave length, method detection, quantification limit, correlation coefficient, and calibration curve equations are given in Table 2. From the correlation coefficients, it is possible to say that the change in absorbance with the concentration of each metal was in a good positive correlation and linearly fit.

**Table 2:** The wavelength used with ICP-OES, instrumental detection limit (IDL), limit of detection (LOD), limit of quantification (LOQ), correlation coefficient, and calibration equations

Metal	Wavelength	IDL (mg/L)	LOD (mg/L)	LOQ (mg/L)	Correlation coefficient	Calibration equation
Na	589.592	0.069	2.16	7.22	0.9988	I = 9245C-7006.1
Mg	285.213	0.002	0.55	1.83	0.9999	I = 1575000C-287546.9
Ca	317.933	0.010	2.92	9.75	0.9990	I = 641100C-632445.1
Mn	257.610	0.001	0.54	1.79	0.9990	I = 4813000C-858583
Fe	238.204	0.004	0.67	2.25	0.9996	I = 590900C-131479.4
Cr	267.716	0.007	0.44	1.48	0.9991	I = 640200C-92120
Co	228.616	0.007	0.31	1.03	0.9994	I = 209100C-20260.1
Ni	231.604	0.015	0.39	1.30	0.9994	I = 185200C-20700.7
Cu	327.393	0.009	0.25	0.82	0.9996	I = 822900C-66599.7
Zn	206.200	0.005	0.75	2.50	0.9980	I = 96750C-24088.4
Cd	228.802	0.030	0.15	0.51	0.9995	I = 353000C-14855.8
K	766.490	–	6.78	21.3-	0.9968	I = 4614C + 3950.7

*I is emission intensity and C is concentration

### Statistical data analysis

Multivariate analysis methods used in this study included one-way analysis of variance (ANOVA), principal component analysis (PCA), linear discriminant analysis (LDA), which were performed with software SPSS 25.0 (SPSS Inc., Chicago, USA) and SIMCA 13 (Umetrics, Sweden). One-way ANOVA was used to test the presence of significant differences in the mean concentration of metals among samples. Duncan’s multiple comparisons was carried out to compare mean values of elements among samples. Differences were considered as statistically significant when p < 0.05. Principal component analysis (PCA) was used to reduce data dimensionality, to visualize sample trends and identify the most discriminating elements among samples [23, 24]. Linear discriminant analysis (LDA) was used to construct classification models useful to predict the geographical origin of teff samples.

## Results and discussion

### Analytical characteristics of the method

The efficiency of the analytical method was verified by testing its accuracy, sensitivity, repeatability, and linearity. The accuracy of the optimized digestion procedure was evaluated by analyzing the digests of spiked samples with standard solutions of each metal. The percentage recoveries varied in the range 85.5–109%, which signifies that the method possesses good accuracy.

### Concentration of metals in teff samples

The teff samples collected from 21 sub-districts in three administrative zones had variable concentrations of metals, as indicated in Table 3. The concentration of K was the highest followed by Mg and Ca,while Cd, Co, and Cr were the lowest in concentration. The concentration (mg/kg) of metals in the teff samples ranged 1746.9–4850.2 K, 159.8–291.6 Na, 1391.7–3403.1 Mg, 951.6–2985.5 Ca, 0.6–17.7 Cr, 25.9–337.4 Mn, 94.6–900 Fe, 0.1–9.9 Co, 1.2—15.6 Ni, 4.7–16.7 Cu, 25.5–187.3 Zn, and 0.1–4.1 Cd.

**Table 3:** The concentration of metals (mean ± standard deviation) in mg/kg dry weight in teff samples from 21 sub-districts of three zones

Zones	Districts	Sub-districts	Varieties of teff	No of samples	Na	K	Mg	Ca	Cr	Mn	Fe	Co	Ni	Cu	Zn	Cd
East Gojjam	Aneded	Jama didik	Kuncho	n = 3	235.1 ± 11.6	2116.5 ± 319.6	1649.6 ± 155.7	1135.0 ± 75.4	17.7 ± 0.03	259.1 ± 18.8	175.2 ± 135.2	9.9 ± 0.1	15.6 ± 1.5	16.7 ± 1.2	53.2 ± 2.9	4.1 ± 0.99
		Guadalema	Kuncho	n = 3	196.6 ± 37.2	2226.3 ± 86.54	1742.3 ± 269.7	1202.8 ± 49.0	12.9 ± 9.9	208.7 ± 120.9	199.3 ± 96.0	3.5 ± 5.5	9.6 ± 9.2	10.3 ± 5.0	34.1 ± 14.0	1.4 ± 0.02
		Adisgeyegewera	Kuncho	n = 3	289.6 ± 79.2	4850.2 ± 2736.4	2997.8 ± 1177.3	1453.8 ± 119.3	2.1 ± 1.7	337.4 ± 185	164.9 ± 129.4	0.3 ± 0.1	4.5 ± 2.0	9.8 ± 3.3	32.4 ± 9.3	ND
	Shebelbernta	Gedaiyasus	Kuncho	n = 6	272.5 ± 72.1	4416.2 ± 1926.6	2149.7 ± 557.1	1232.8 ± 85.6	2.5 ± 1.9	31.2 ± 6.0	94.6 ± 59.1	0.1 ± 0.03	2.0 ± 0.6	8.6 ± 1.6	25.5 ± 7.1	ND
		Yeidwuha Town	Kuncho	n = 3	236.1 ± 24.3	4305.9 ± 880.9	1999.6 ± 251.5	1170.4 ± 58.2	1.9 ± 1.7	27.0 ± 2.8	95.1 ± 15.3	0.1 ± 0.03	1.6 ± 0.7	7.1 ± 1.1	39.7 ± 2.5	ND
		Weregona akababiw	Kuncho	n = 3	261.2 ± 41.4	3709.6 ± 627.9	2055.8 ± 200.9	1170.6 ± 121.1	0.6 ± 0.6	34.5 ± 11.0	128.5 ± 56.1	0.1 ± 05	1.2 ± 0.1	5.6 ± 0.2	111.9 ± 98.0	0.1 ± 0.01
	Enamey	Weyira gurazem	Kuncho	n = 3	205.8 ± 34.3	2231.3 ± 88.7	1391.7 ± 46.9	1714.7 ± 351.7	10.5 ± 0.7	30.0 ± 3.3	272.2 ± 111.8	0.7 ± 0.11	7.4 ± 0.5	5.0 ± 1.5	32.2 ± 5.0	1.3 ± 0.01
		Hulet amibadibisa	Kuncho	n = 3	204.3 ± 47.2	2443.3 ± 293.0	1570.0 ± 111.3	1555.0 ± 163.3	11.7 ± 1.7	42.7 ± 0.9	249.6 ± 32.0	2.1 ± 1.1	10.1 ± 2.4	4.7 ± 1.2	55.9 ± 20.1	2.2 ± 0.02
		Enerkornaadis amba	Kuncho	n = 3	213.6 ± 35.0	2178.3 ± 67.3	1629.0 ± 458.3	1352.0 ± 330.0	11.3 ± 1.5	56.9 ± 24.7	248.2 ± 136.7	2.4 ± 1.4	10.8 ± 3.3	5.6 ± 1.6	70.4 ± 35.2	2.7 ± 1.2
West Gojjam	GoinjiKolela	Zanat	Kuncho	n = 3	248.7 ± 68.6	3926.4 ± 260.3	2317.2 ± 97.1	951.6 ± 93.9	1.4 ± 1.3	25.9 ± 2.1	138.6 ± 27.2	0.3 ± 0.06	1.2 ± 0.2	5.8 ± 0.4	65.0 ± 23.3	0.1 ± 0.09
		Gonj	Kuncho	n = 3	225.5 ± 53.7	2881.7 ± 507.4	2288.3 ± 276.1	1025.2 ± 102.3	1.4 ± 0.4	56.0 ± 25.7	166.0 ± 52.6	0.6 ± 0.07	1.6 ± 0.1	5.9 ± 0.7	46.4 ± 8.2	0.2 ± 0.05
		Kore Tenkere	Kuncho	n = 3	185.3 ± 4.5	2604.0 ± 446.7	2117.2 ± 105.5	1257.1 ± 125.9	2.0 ± 1.2	73.3 ± 21.2	157.0 ± 50.4	0.8 ± 0.08	2.1 ± 0.5	5.4 ± 0.8	54.1 ± 25.0	0.4 ± 0.08
		Menta Deber	Kuncho	n = 3	197.2 ± 28.6	2316.5 ± 112.3	2135.6 ± 271.2	1206.5 ± 50.4	1.7 ± 0.2	72.6 ± 23.3	184.5 ± 87.7	1.1 ± 0.1	2.5 ± 0.3	5.0 ± 0.8	41.7 ± 6.1	0.7 ± 0.15
	Adet	Kilelet	Kuncho	n = 3	184.6 ± 25.9	1852.4 ± 123.0	2088.4 ± 246.7	1344.1 ± 211.1	1.8 ± 0.9	88.9 ± 17.8	263.3 ± 82.6	1.5 ± 0.1	2.9 ± 0.3	5.0 ± 0.3	56.1 ± 6.7	0.8 ± 0.01
		Adet Zuria	Kuncho	n = 2	159.8 ± 6.5	1842.6 ± 84.9	2306.8 ± 39.0	1632.9 ± 118.9	4.2 ± 2.4	162.8 ± 42.7	269.0 ± 76.1	1.8 ± 0.6	5.4 ± 2.1	6.1 ± 0.01	70.2 ± 8.5	1.1 ± 0.01
		Mosbo	Kuncho	n = 4	225.9 ± 33.5	1746.9 ± 98.6	2369.1 ± 241.7	1949.0 ± 132.8	3.7 ± 1.0	178.3 ± 67.7	515.5 ± 203.9	2.2 ± 0.1	5.8 ± 0.5	6.1 ± 0.8	80.3 ± 19.3	1.3 ± 0. 1
Awi	Zigam	Gisayita	Sergegna	n = 3	188.8 ± 33.2	1967.8 ± 193.7	2484.1 ± 1043.5	2031.7 ± 622.0	5.6 ± 3.6	241.3 ± 71.0	791.5 ± 494.3	1.2 ± 0.04	5.1 ± 3.5	5.9 ± 0.9	62.3 ± 17.3	0.5 ± 0.01
			Qey	n = 3	291.6 ± 21.9	2880.3 ± 422.5	2972.1 ± 730.3	2461.2 ± 344.0	4.4 ± 2.5	222.0 ± 78.6	744.5 ± 473.8	0.5 ± 1.3	4.0 ± 1.4	6.6 ± 1.1	80.0 ± 24.9	0.0 ± 0.02
			Tseday	n = 3	286.8 ± 46.7	3823.7 ± 1750.4	2536.4 ± 575.5	2615.5 ± 833.0	3.1 ± 1.7	272.7 ± 15.3	614.7 ± 122.5	0.7 ± 0.2	3.7 ± 0.8	5.8 ± 0.6	86.3 ± 17.9	0.1 ± 0.02
		Zigam Town	Tseday	n = 3	224.2 ± 28.9	3202.9 ± 90.8	3403.1 ± 1343.0	2946.5 ± 1600	4.7 ± 5.7	222.0 ± 85.7	637.1 ± 332.0	0.9 ± 0.7	4.7 ± 3.5	6.5 ± 1.5	116.6 ± 76.0	0.2 ± 0.01
		Ahiti	Tseday	n = 3	225.7 ± 41.6	2583.9 ± 190.5	2451.9 ± 423.0	2679.8 ± 226.6	3.5 ± 1.4	235.1 ± 90.0	376.8 ± 126.5	0.9 ± 0.2	4.7 ± 0.2	5.3 ± 1.1	103.5 ± 24.7	0.3 ± 0.01
		Gagasta	Tseday	n = 3	172.8 ± 29.9	1865.3 ± 41.0	2087.5 ± 248.8	2582.8 ± 228.5	6.3 ± 1.3	195.4 ± 25.1	900.0 ± 163.8	1.7 ± 0.4	6.6 ± 1.3	4.7 ± 0.5	84.3 ± 12.0	0.5 ± 0.1
		GudarJawi	Tseday	n = 3	175.8 ± 7.07	2434.0 ± 65.198.6	3132.6 ± 101.85	2985.5 ± 229.9	4.9 ± 0.7	178.9 ± 17.5	575.9 ± 177.5	2.6 ± 0.4	7.9 ± 2.1	5.0 ± 0.4	187.3 ± 63.6	1.0 ± 0.2

### Multi-elements variation among different regions

In general, the pattern of concentration of metals in teff samples from the three administrative zones was in decreasing order of K > Mg > Ca > Fe > Na > Mn > Zn > Cu > Cr > Ni > Co > Cd. The concentrations of Fe, Mn, Zn, Cu, Ca, Co, Mg, Na, and K in this study were in agreement with the previous studies [25–27]. The concentration of K was the most abundant element, followed by Mg and Ca in teff samples corroborated with the previous studies [26, 28–30]. A similar trend, i.e., a higher concentration of K, has also been reported in teff grains from other areas of Ethiopia [31]. The high concentrations of K, Ca, and Mg in teff grains could be due to the fact that these elements are readily available from the soil to the plant to translocate into the different parts of the plant [31, 32].

Regarding teff grains from the three zones, the mean concentrations of K and Na were highest in samples from East Gojjam, followed by Awi zone. Samples from the Awi zone were rich in the mean concentration of Mg, Ca, Fe, and Zn followed by samples from the West Gojjam zone (Table 4). The mean concentrations of the major elements in the teff samples were in the order of K > Mg > Ca > Na (Table 4). Similarly, the mean concentrations of trace elements in teff grains from Awi and West Gojjam zones were ordered as Cu > Ni > Cr > Co > Cd. Whereas the comparative order of these metals in samples from the East Gojjam zone was recorded as Cu > Cr > Ni > Co > Cd. This indicates the uptake of metals by plants takes place through different and complex biochemical processes. The uptake processes vary based on the nature of the metal, ability of the plants to absorb metals from the soil, the availability of the minerals in soluble and usable forms, the abundance of particular minerals in the particular areas, and the degree of contamination of the soil with heavy metals.

**Table 4:** Mean, maximum (Max), minimum (Min) concentration (mg/kg) and standard error (SE) the elements determined in teff grains from three zones of Amhara Region, Ethiopia

Zone		Na	K	Mg	Ca	Cr	Mn	Fe	Co	Ni	Cu	Zn	Cd
East Gojjam	Mean	234.9 ^a^	3164.1^a^	1909.5^b^	1331.9^b^	7.9^b^	114.1^a^	180.8^b^	2.1^a^	6.9 ^a^	8.1^a^	50.5^a^	1.3^a^
	Max	289.6	4850.2	2997.8	1714.7	17.7	337.4	272.2	9.9	15.6	16.7	111.9	4.1
	Min	196.6	2116.5	1391.7	1135	0.6	27	94.6	0.1	1.2	4.7	25.5	0
	SE	11	379.1	159.3	67.5	2.1	40.1	22.2	1.1	1.6	1.2	9	0.4
West Gojjam	Mean	203.8 ^a^	2452.9 ^a^	2231.8 ^b^	1338.1 ^b^	2.3 ^a^	93.9 ^a^	241.9 ^b^	1.2 ^a^	3.1 ^a^	5.6 ^a^	59.1 ^a^	0.6 ^a^
	Max	248.7	3926.4	2369.1	1949	4.2	178.3	515.5	2.2	5.8	6.1	80.3	1.3
	Min	159.8	1746.9	2088.4	951.6	1.4	25.9	138.6	0.3	1.2	5 .00	41.7	0.1
	SE	11.6	293.9	43.1	131.9	0.4	21.2	49.5	0.2	0.6	0.2	5.1	0.2
Awi Zone	Mean	223.6 ^a^	2679.7^a^	2723.9 ^a^	2614.7 ^a^	4.6 ^a^	223.9^b^	662.9 ^a^	1.2 ^a^	5.2 ^a^	5.6 ^a^	102.9 ^b^	0.3 ^a^
	Max	291.6	3823.7	3403.1	2985.5	6.3	272.7	900	2.6	7.9	6.6	187.3	1
	Min	172.8	1865.3	2087.5	2031.7	3.1	178.9	376.8	0.5	3.7	4.7	62.3	0
	SE	18.7	261.1	173.3	121.1	0.4	11.6	63.9	0.27	0.5	0.2	15.5	0.1

Statistical analysis with one-way ANOVA (α = 0.05) was used to test the presence of significant differences in the mean elemental contents of teff from the three administrative zones (Table 4). Samples from Awi zone contained significantly higher mean concentrations of Mg, Ca, Fe, and Zn than teff samples from East Gojjam and West Gojjam zones. On the other hand, no statistically significant difference was observed between the samples from East Gojjam and West Gojjam zones in their mean concentrations of Mg, Ca, Fe, and Zn. Concerning the other elements, except Mn and Cr, no significant difference was observed in the levels of Na, K, Ni, Cu, Cd, and Co among samples from the three zones. Significantly higher concentration of Mn was found in teff grains from Awi zone, while higher Cr in teff grains from East Gojjam. The observed significant variation among production zones might be due to the differences in the growing environmental conditions and crop inherent potential to take nutrients from the soil, resulting in differences in their metabolic activities like accumulations of different metals in their body [9, 33, 34].

The variations in the metal contents of teff grains from the different sub-districts was assessed using the standard deviation (SD) values (Table 3). Most of the sub-districts have been found to show less variation in their metal contents, which indicated that the sampling areas within a given sub-districts have similar agroecological conditions. Whereas, a higher variation was noted for some of the sub-districts, which indiced the existence of different agroecological conditions within the sub-districts. Some reports has indicated that variations in mineral contents in plants grown in different areas occurs when factors such as agricultural practices, soil chemistry, and plant genotype are varied [9, 33, 34].

### Multi-elements variation among different varieties

Comparing the four varieties of teff collected from the three zones,Tseday teff variety was found to be rich in Mg (3403 mg/kg), Ca (2986 mg/kg), Fe (900 mg/kg), and Zn (187 mg/kg). The concentration of K (4850 mg/ kg) was highest in Kuncho teff variety when compared with the others (Table 3). The differences in the concentration levels of metals in varieties of teff arise mainly due to differences genetic properties, soil type, agricultural practices, and other environmental factors [31]. In the Amhara region, some farmers in some district use crop rotation systems to improve soil fertility, while other districts constantly cultivate the same crop type for many years at the same place [35]. Secondly, the soil types of the three sampling zones are different. Most East Gojjam teff samples are grown in black soil, while others are produced in grey and red soils [36, 37].

The concentrations of the trace elements (Cu, Zn, Co, Ni, Mn, and Cr) are in good agreement with the results reported by Habte et al. [26] and Baye et al. [38], as indicated in Table 5. Similarly, the determined concentrations of Mn and Cu lies within the range of values reported by Gebregewergis [39] and Habte et al. [26]. On the other hand, the determined concentrations of Ca and Zn are higher than that reported by Baye et al.[38], and the amounts of Co, Cu, and Cr were higher than that reported by Dame [28]. The literature generally indicates that the amount of metals in teff grains varies considerably with geographical origin, which can be attributed to variations in growing soil types, environmental conditions, and other factors, as it is also supported by previous findings of other researchers [31, 33, 34].

**Table 5:** Elemental composition (mg /kg dry weight) of teff samples reported in the literature

Types of teff	Mn	Fe	Zn	Cu	Ca	Cd	Co	Mg	Na	K	Cr	Ni	References
White	21.2	196	67	520	–	–	–	1080	–	–	–	–	[40]
Purple	30	115	68	640	–	–	–	–	–	–	–	–	
White	64	58	20	7	1590	–	–	1070	471	4010	–	–	[29]
Qey		116–1500	23–67	11–36	180–1780	–	–	–	–	–	–	–	[38]
White		95–377	24–68	25–53	170–1240	–	–	–	–	–	–	–	
Sergegna		115–1500	38–39	16	788–1470	–	–	–	–	–	–	–	
Qey	20	1195	80	15	348	0.8	–	–	–	–	–		[39]
White	21	485	85	13	266	1.3	–	–	–	–	–		
Sergegna	28	645	73	13	247	1.8	–	–	–	–	–	–	
White	484	1595	298	108	1807	-	78.9	1531.6	228.9	2055.9			[27]
White	–	–	–	–	1560	–	–	–	–	–	–	–	[6]
Brown	–	–	–	–	1570	–	–	–	–	–	–	–	
Sergegna		589	–	–	–	–	–	–	–	–	–	–	
Brown		820	180	–	1480	–	–	1699	290	3535	–	–	[30]
White	70	212	46	7.5	1320	–	–	2479	84	2883	–		[26]
Brown	68	214	38	6.7	1211	–	–	2469	88	2948	–	–	
Qey	58	232	85	16	1739	–	–	2552	90	3071	–	–	
White	–	145.5	–	–	–	–	–	–	–	–	–	–	[5]
White	–	11.4	9.58	1.65	315	-	0.05	543	127.85	1289	0.71	0.69	[28]
White	–	4	–	–	1	–	–	–	–	–	–	–	[9]
white	-	915	233		2056.1	–	–	–	–	–	–	–	[25]
Brown	-	931	230		1973.2	–	–	–	–	–	–	–	
Kuncho	25.9–337.4	128.5–515.5	25.5–80.3	4.7–16.7	1135–1949	0.1–4.1	0.1–9.9	1391.7–2369.1	159.8–289.6	1746.9–4850.2	0.6–17.7	1.2–15.6	This study
Tseday	178.9–272.7	376.8–900	84.3–187.3	4.7–6.5	2582.8–2985.5	0.1–1.0	0.7–2.6	2087.5–3403.1	172.8–286.8	1865.3–3823.7	3.1–6.3	3.7–7.9	
Qey	222	744.5	80.0	6.6	2461.2	-	0.5	2972.1	291.6	2880.3	4.4	4.0	
Sergegna/Brown	241.3	791.5	62.3	5.9	2031.7	0.5	1.2	2484.1	188.8	1967	5.6	5.1	

^*^Different letters in a column indicates that the values are significantly different at p > 0.05

### Principal component analysis

**Fig. 2:**
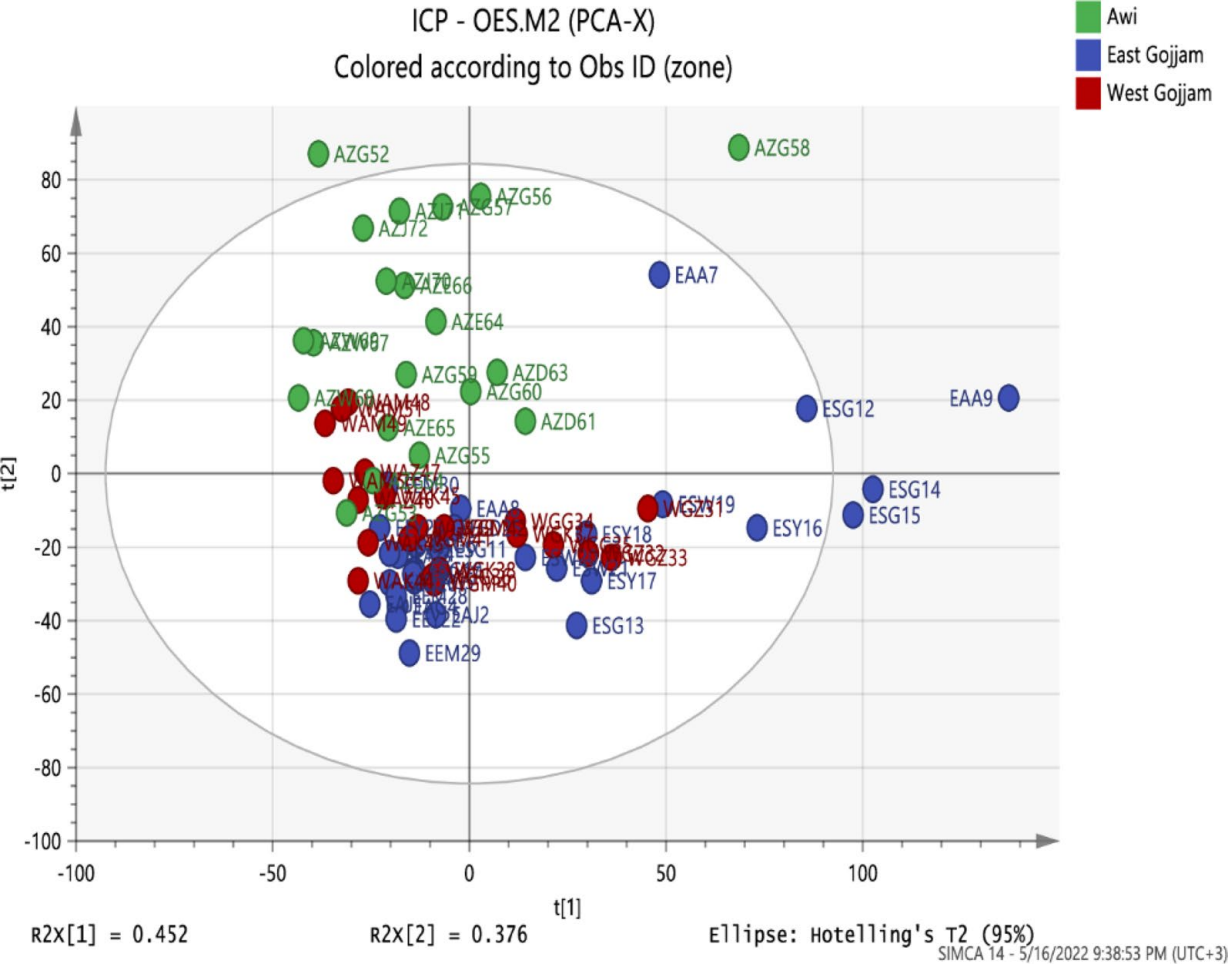
Score plot of the first two principal components of the principal component analysis model, indicating the distribution of teff samples from three administrative zones of Amhara Region, Ethiopia

**Fig. 3:**
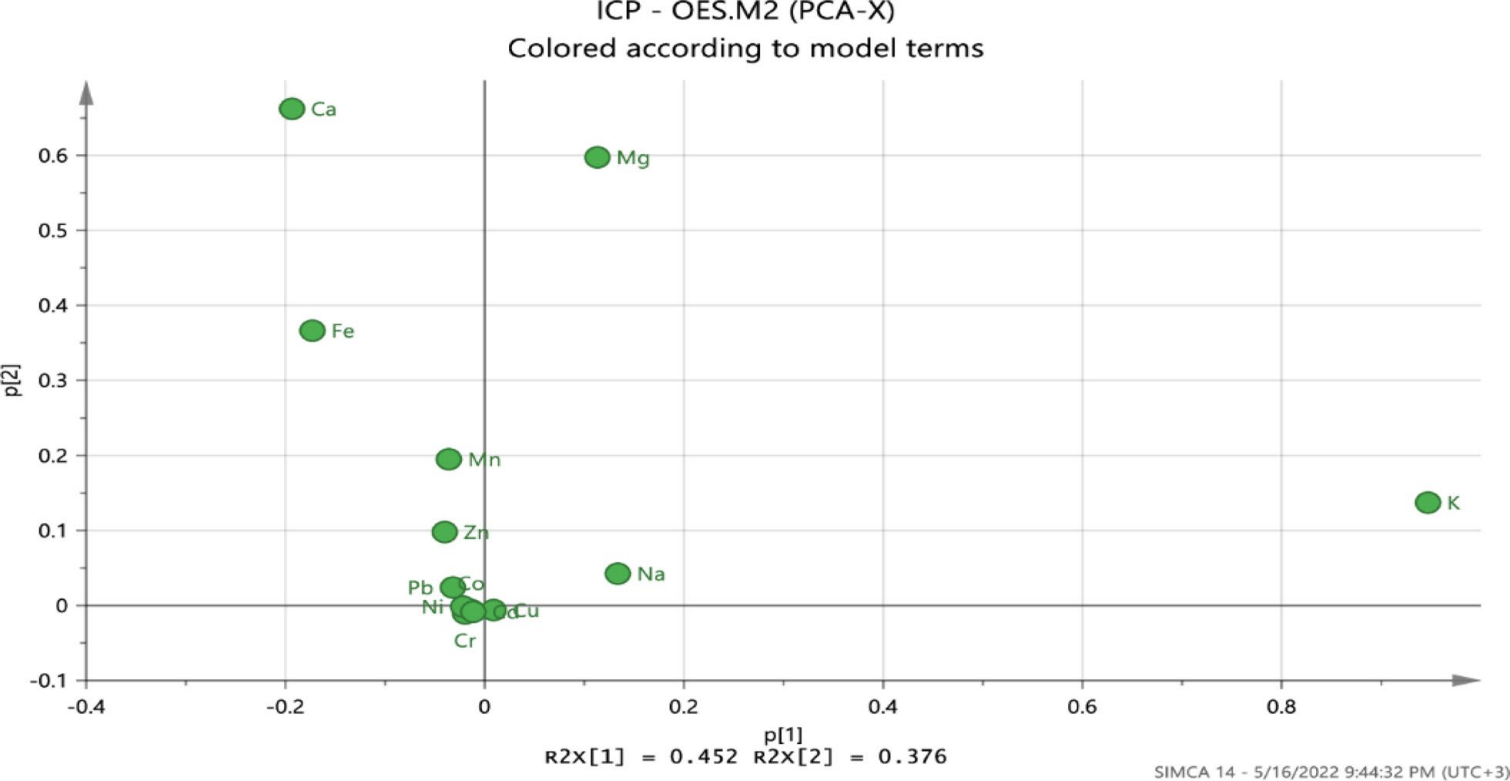
Loadings plot for the first two principal components obtained from principal component analysis metals in teff grains from three administrative zones of Amhara Region, Ethiopia

A preliminary investigation based on principal component analysis (PCA) was applied to visualize sample trends and evaluate the discriminatory characteristics of the determined elements. The data set was subjected to different scaling tools (Pareto, SNV, UV, freeze, and others) using the statistical software tool SIMCA 13 (Umetrics, Sweden). The Pareto scaling was found to provide excellent model parameters (Q2 (cum) = 0.568 and R2x (cum) = 0.922 with two principal components). Together, the first component (PC1) and second component (PC2) explained 82.8% of the variation in the data. The score plots and loading plots are shown in Figs. 2 and 3, respectively. The separation of the teff samples by the first two components is illustrated by the scores plot in Fig. 2, which indicates the presence of groups and some other important patterns in the data. The teff samples from Awi zone are clustered from the other zone teff samples and lie on the positive side of PC2, where two of the samples were found to be outliers. Teff samples from East Gojjam are presented on the negative side of PC1 and PC2, where two of them are scattered on the positive side of PC1 and three samples were found as outliers. At the same time, teff samples from West Gojjam zone lie on the negative side of PC1 and PC2. The elements responsible for separation in the first component were Cu, Mg,and Na, whereas separation along principal component 2 was influenced primarily by Ni, Ca, and Fe (Fig. 3).

Teff grains from Awi zone were distinguished by their higher concentrations of Ca, Mg, and Fe. These observations are in agreement with the results obtained with one-way ANOVA. Similarly, teff grains from East Gojjam and West Gojjam were separated mainly due to their higher concentrations of Na and K. The loadings plot (Fig. 3) displays how the individual elements correlate with each other and contribute to the PCA model. Elements far away from the origin have a strong impact on the model, whereas those closer to the center have a weaker influence. From the plot, it is evident that K, Mg, Ca, and Fe play the largest role in discriminating the teff samples among the administrative zones. Hence,these elements are potentially good chemical descriptors for the construction of classification models for teff grains from the three zones.

### Linear discriminant analysis at zones and districts

Evaluating the contributions of different factors towards variations in elemental contents of teff grains from different zones and varieties was not within the scope of this study. Instead, the study assumed that the different teff growing zones may have differences in one or more of different factors like local climate (rainfall, temperature, humidity, and solar radiation), soil type and agronomic practices that can result in significant variations in the elemental contents of teff grains cultivated in different areas. Hence, attempt was made to evaluate the adequacy of the overall variations in elemental concentrations for use as descriptors in chemometric models for the geographical origin determination of the grains.

**Fig. 4:**
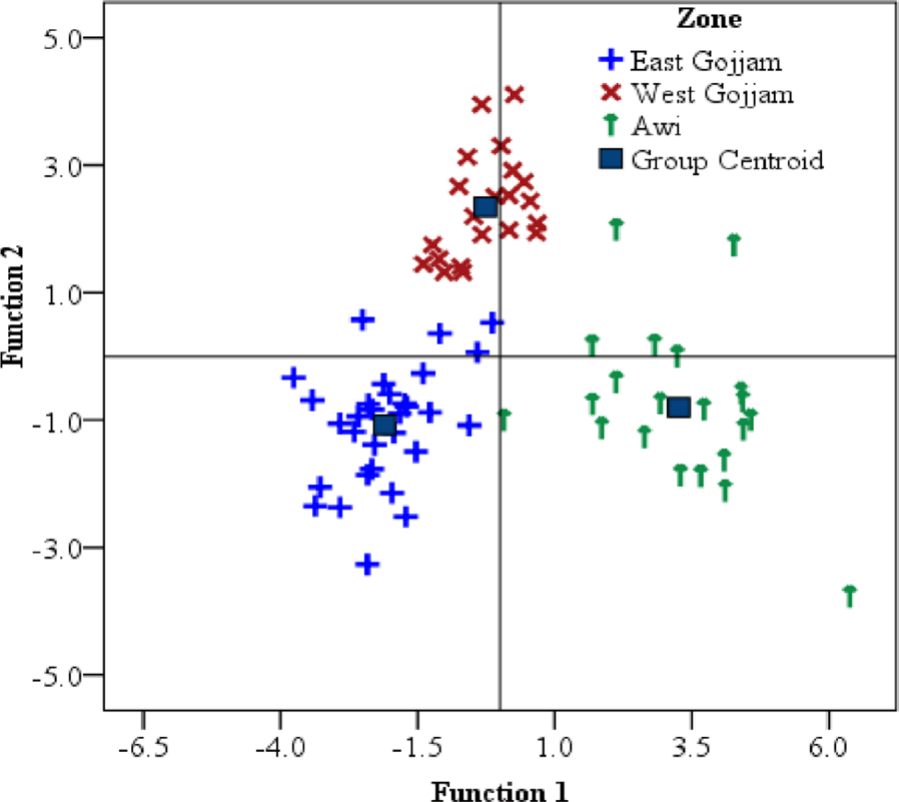
Scatter plot showing the distribution of teff samples on the space created by the scores of the first two discriminant functions

Linear discriminant analysis was applied to construct classification models for teff grains cultivated in the studied three zones. Accordingly, two discriminant functions were computed, where the first accounted for 69% and the second 31% of the total variance present in the data set. The LDA model provided 96% correct classification, where only 3 out of the 72 samples were incorrectly classified. Two samples from Awi zone were incorrectly classified, one as East Gojjam and the other as West Gojjam, as well as one sample from East Gojjam was incorrectly classified as from West Gojjam. The separation between the geographical origins of teff samples in the discriminant space was examined by plotting two function scores in Fig. 4. It was clearly observed that samples from the three administrative zones were well distinguished from each other, demonstrating that elements contained enough information to identify the zones where the teff samples were grown.

The distribution of the teff samples on the plane of the two functions is shown in Fig. 4. The first function effectively distinguished between East Gojjam and Awi zone teff samples. This function is correlated mainly with Ca and Fe, followed by Mg, Zn, and Mn (Table 6). Teff samples from Awi zone occupy the space to the positive side of the first function, hence characterized by higher contents of Ca and Fe than samples from East Gojjam.The second function distinguished West Gojjam from East Gojjam and Awi zone teff samples. This function is correlated mainly and negatively with Cr and Ni, followed by Cu, K, and Na in Table 6. Teff samples from West Gojjam occupy the space to the positive side of the second function, hence characterized by lower contents of Cr and Ni than samples from East Gojjam and Awi zones. Hence, the Ca, Fe, Mg, and Zn were the main discriminating factors among teff samples from Awi zone Table 6.

**Table 6:** Canonical discriminant function coefficients of the linear discriminant model constructed with the metal contents of teff grain samples from three administrative zones of Amhara Region, Ethiopia

**Element**	Ca	Fe	Mg	Zn	Mn	Cd	Cr	Ni	Cu	K	Na	Co
Function 1	0.54	0.41	0.25	0.24	0.24	− 0.13	− 0.09	− 0.04	− 0.16	− 0.08	− 0.04	− 0.05
Function 2	− 0.27	− 0.11	0.01	− 0.05	− 0.16	− 0.06	− 0.25	− 0.22	− 0.19	− 0.15	− 0.15	− 0.06

The reliability of using the determined elements as descriptors with LDA modeling, in developing an analytical method for determining the origin of teff from the three zones, was evaluated from the prediction ability of the model. For this, randomly selected 24 samples, out of the total 72 samples, were used as a validation set, while the remaining 48 samples were used as a training set to construct the LDA model. The analysis was carried out five times by changing the 24 random samples, and the average of the five iterations was calculated. Consequently, the model provided a prediction ability of 91.7%, equivalent to two samples out of the 24 samples whose group membership was incorrectly predicted.

Additionally, leave-one-out cross-validation analysis was performed, where each sample was classified by the LDA model constructed with the remaining sample set. In this, 91.7% of the cross-validated samples were correctly classified.

Regarding the growing districts, application of LDA provided 93.1% correct classification of the teff grain samples into the respective growing districts. Only 5 from the total 72 samples were incorrectly classified. These were, 2 samples from Aneded, one incorrectly classified as Shebelberenta and the other as Gonjikolela, one sample from Shebelberenta, which was incorrectly classified as Gonjikolela, and one sample from Zigam, which was incorrectly classified as Shebelberenta.

**Fig. 5:**
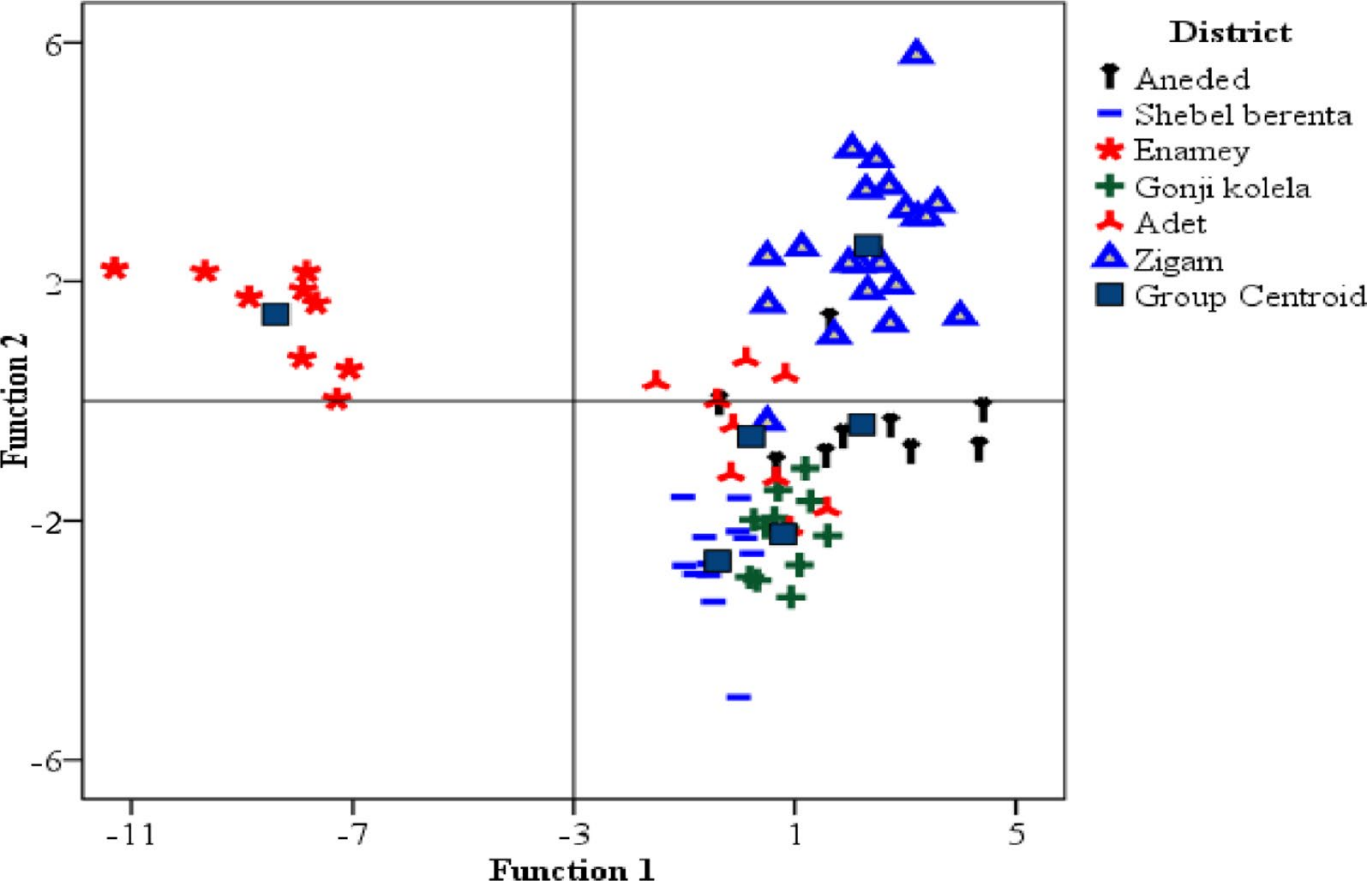
Scatter plot of teff samples from six districts on the plane of the first two discriminant functions

Three clusters were evident from the LDA scores plot of teff grains from the six districts (Fig. 5). These are teff grains from Zigam, Enamey and a group of samples from Shebelberenta, Gonjikolela and Adet. Enamey is discriminated from the other districts by the first function, which explained 53% of the data variance. Magnesium is more associated with this function (Table 7). Hence, teff from Enamey is differentiated by its higher content of Mg. Teff from Zigam is discriminated from the remaining districts by the second function, which accounted for 21% of the data variance.The elements Ca, Mn, Fe, and Zn are correlated with the second function (Table 7). Hence, Ca, Mn, Fe, and Zn were good descriptors of teff samples from Zigam district.

**Table 7:** Correlation between the analyzed elements and discriminant functions

Element		Mg	Ca	Mn	Fe	Zn	Cu	Ni	Cr	K	Cd	Co	Na
1		0.16	0.13	0.28	0.11	0.06	0.10	− 0.10	− 0.12	0.03	− 0.13	0.01	0.03
2		0.13	0.62	0.45	0.44	0.20	− 0.14	0.27	0.19	− 0.17	0.08	0.06	− 0.06
3		0.10	0.19	− 0.33	0.17	0.13	− 0.58	− 0.38	− 0.37	− 0.11	− 0.26	− 0.28	− 0.11
4		− 0.05	− 0.12	0.32	− 0.00	− 0.05	0.11	0.28	0.15	− 0.37	0.34	0.293	− 0.19
5		− 0.06	− 0.36	0.03	− 0.16	− 0.13	− 0.04	0.16	0.21	− 0.19	0.21	0.168	− 0.19

### Linear discriminant analysis of teff varieties

**Fig. 6:**
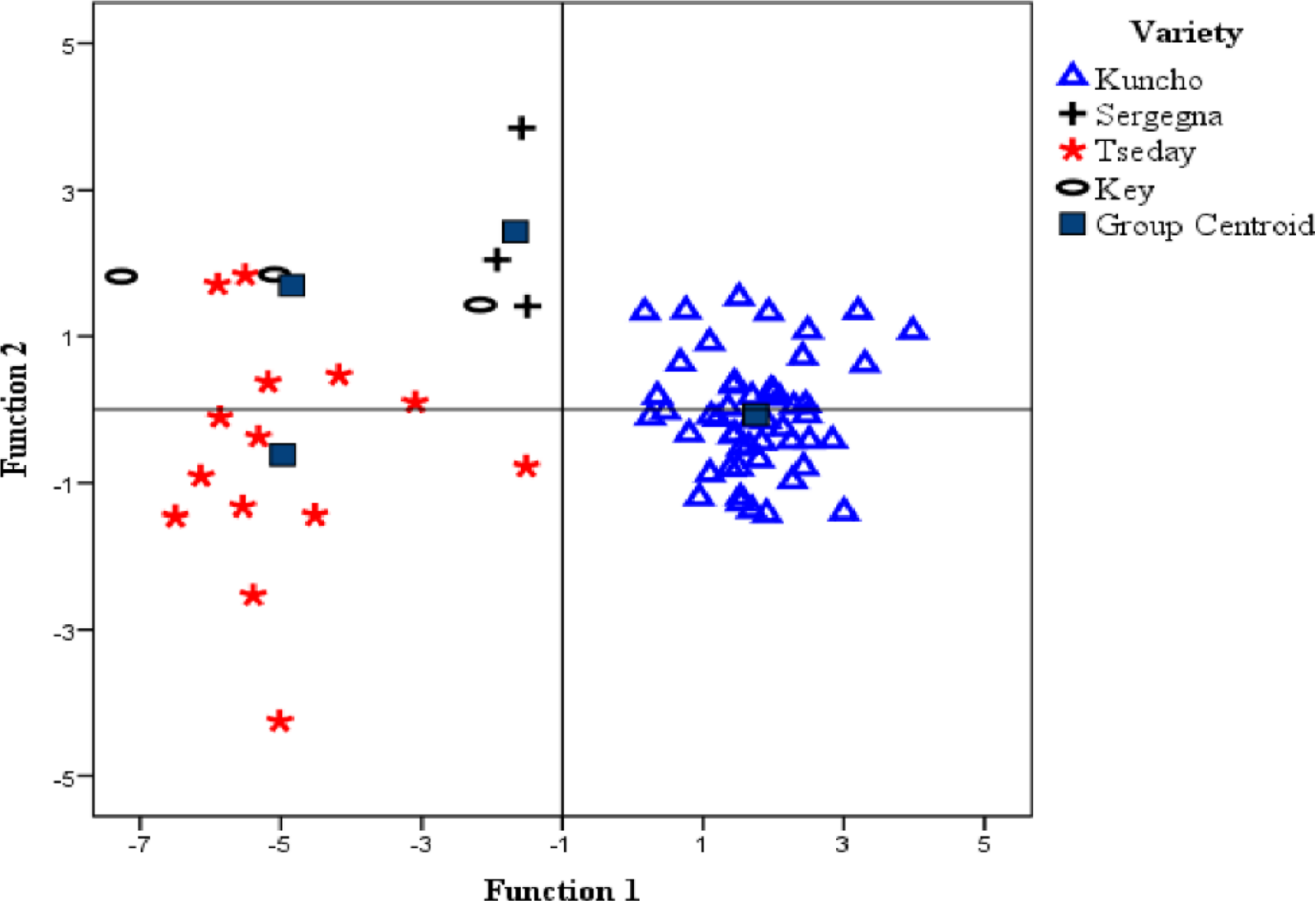
Scatter plot of four varieties of teff samples on the space of the first two discriminant functions

The potential of using the elemental concentrations in the construction of discriminant models useful for the authentication of the four varieties of teff (Kuncho, Tseday, Key, Sergegna) was also investigated. From the computed three discriminant functions, the first two accounted for 93% of the variation in the data set.The LDA model provided 93% correct classification of the 72 teff grain samples into the four varities. Where, 4 samples of Tseday variety were incorrectly classified, 3 as Key and one as Kuncho, and one sample of Key variety incorrectly classified as Sergegna. Distinct discrimination between Kuncho and Tseday varieties was observed by the first function (Fig. 6), which accounts for 92.6% of the total variance in elemental concentrations among varieties of teff. The elements Ca, and Fe were the highest contributors to the first function, and Tseday variety was distinguished from Kuncho by its higher content of Ca and Fe (Table 8). Hence, Ca and Fe were the main discriminating factors among the teff samples depending on varieties.

**Table 8:** Correlation between elements in varietal types of teff samples and discriminant functions

Element		Ca	Fe	Mn	Zn	Mg	Na	Ni	K	Co	Cr	Cu	Cd
Function	1	− 0.53	− 0.3	− 0.19	− 0.19	− 0.16	− 0.01	0	0.03	0.03	0.03	0.08	0.08
	2	0.06	0.46	0.33	− 0.18	0.02	0.06	− 0.07	0.02	− 0.07	0	− 0.02	− 0.1
	3	0	− 0.5	− 0.01	− 0.04	− 0.14	0.64	− 0.13	0.54	− 0.07	− 0.12	0.08	− 0.07

## Conclusion

An efficient digestion procedure for the determination of metals in the teff samples was optimized and validated through the spiking method, and a good percentage recovery was obtained from 85.5 to 109% for all the metals of interest. The levels of metals in teff samples were in the order K > Mg > Ca > Fe > Na > Mn > Zn > Cu > Cr > Ni > Co > Cd. One-way ANOVA revealed the presence of significant difference (p < 0.05) in the mean concentrations of metals, except for K, Na, Co, Ni, Cu and Cd,among the sampling zones. With the aid of statistical pattern recognition,metals Ca and Fe, followed by Mg, Zn, and Mn were identified as the most discriminating metals for teff samples from three sampling zones. Ca and Fe were selected as suitable discriminant marker for teff samples originating from Awi zone,as well as varietal types of teff. While, K was the marker element for teff from East Gojjam zone. Application of LDA successfully classified the teff samples in to three administrative zones. The LDA model provided a prediction ability of 91.7%, equivalent to two samples out of the 24 samples whose group membership was incorrectly predicted. Additionally, leave-one-out cross-validation analysis of the entire 72 samples resulted in 91.7% correct classification. Hence, multielement data combined with LDA modeling can be used in the authentication of the geographical origin and varietal types of teff from Amhara region.

## Data Availability

The datasets used and/or analysed during the current study are available from the corresponding author on reasonable request.
